# Large area scanning probe microscope in ultra-high vacuum demonstrated for electrostatic force measurements on high-voltage devices

**DOI:** 10.3762/bjnano.6.258

**Published:** 2015-12-28

**Authors:** Urs Gysin, Thilo Glatzel, Thomas Schmölzer, Adolf Schöner, Sergey Reshanov, Holger Bartolf, Ernst Meyer

**Affiliations:** 1 Department of Physics, University of Basel, Klingelbergstrasse 82, CH-4056 Basel, Switzerland; 2 ABB Corporate Research Center, Segelhofstrasse 1K, CH-5404 Baden-Dättwil, Switzerland; 3 Ascatron AB, Electrum 207, SE-16440 Kista, Sweden

**Keywords:** copper alloy, electrostatic force microscopy, high-voltage device, Kelvin probe force microscopy, silicon carbide (SiC), surface photo voltage

## Abstract

**Background:** The resolution in electrostatic force microscopy (EFM), a descendant of atomic force microscopy (AFM), has reached nanometre dimensions, necessary to investigate integrated circuits in modern electronic devices. However, the characterization of conducting or semiconducting power devices with EFM methods requires an accurate and reliable technique from the nanometre up to the micrometre scale. For high force sensitivity it is indispensable to operate the microscope under high to ultra-high vacuum (UHV) conditions to suppress viscous damping of the sensor. Furthermore, UHV environment allows for the analysis of clean surfaces under controlled environmental conditions. Because of these requirements we built a large area scanning probe microscope operating under UHV conditions at room temperature allowing to perform various electrical measurements, such as Kelvin probe force microscopy, scanning capacitance force microscopy, scanning spreading resistance microscopy, and also electrostatic force microscopy at higher harmonics. The instrument incorporates beside a standard beam deflection detection system a closed loop scanner with a scan range of 100 μm in lateral and 25 μm in vertical direction as well as an additional fibre optics. This enables the illumination of the tip–sample interface for optically excited measurements such as local surface photo voltage detection.

**Results:** We present Kelvin probe force microscopy (KPFM) measurements before and after sputtering of a copper alloy with chromium grains used as electrical contact surface in ultra-high power switches. In addition, we discuss KPFM measurements on cross sections of cleaved silicon carbide structures: a calibration layer sample and a power rectifier. To demonstrate the benefit of surface photo voltage measurements, we analysed the contact potential difference of a silicon carbide p/n-junction under illumination.

## Introduction

Scanning probe microscopy (SPM) is nowadays an established technological approach for surface analysis in many different research fields. Applications can be found in areas of life science measuring the properties of cells in buffer solution, submolecular structure of single molecules in ultra-high vacuum (UHV) conditions but also in areas which face the characterization of semiconductor devices. The common technical principle is always related to a conical tip attached to a cantilever which is accurately positioned at the specimen of interest and which is scanned over a certain surface area. The tip height is controlled by a feedback loop correlating the tip–sample interaction with the deflection of the cantilever. However, the interaction force contains many different components which can only be partly suppressed (e.g., magnetic forces when inspecting non-magnetic materials), separated (e.g., electrostatic forces from magnetic forces), or be dynamically compensated (e.g., by tuning the bias voltage in Kelvin probe force microscopy (KPFM)) and measured together with the topological information. For all these properties various experimental approaches have been proposed, successfully demonstrated, and found their way into commercially available SPM systems. However, the unperturbed measurements and the interpretation of the acquired data remains the most challenging task which requires a sophisticated fundamental interpretation.

In recent years, especially the detection of electrostatic forces and the determination of local work function values was intensively discussed and models combining large scale influences with atomistic simulations have been developed [1–4]. As early as in the late 1980s H. Wickramasinghe proposed several SPM based methods for the local analysis of the electrical properties of conducting and semiconducting materials down to the nanometre scale [5–12]. These techniques rapidly emerged [13–17] and were developed further on resulting in more sophisticated methods such as scanning spreading resistance microscopy (SSRM) [18–20] and scanning capacitance microscopy (SCM) [21–23]. However, since atomic force microscopy (AFM) [24] was demonstrated to analyse surfaces down to the nanoscale, most of the commercial microscopes are limited to high resolution in UHV or can only be used under ambient conditions. However, for the characterization of complex semiconductor devices large area scans with the possibility of taking high resolution images at dedicated areas under inert conditions are mandatory.

The instrument described in the first part of this article allows for investigations on a scale of up to 100 μm in lateral and 25 μm in vertical direction under UHV conditions and at room temperature using a large-scale closed-loop scanner. Beside the topographic non-contact AFM mode also contact measurements as well as all major electrical characterization methods (SSRM, SCM, KPFM) are implemented. Additionally, the samples can be optically exited by an external light source (UV–vis) which is introduced by a separate light fibre. An in situ piezo-electric alignment stage allows to focus and position the light exactly below the cantilever tip apex. Therefore, the setup allows for the measurement of the surface photo voltage (SPV) in dependence on the wavelength and light intensity via measuring of the contact potential difference (CPD) values in the dark as well as under illumination [25]. In the second part we present several studies highlighting the potential of the novel instrument. Firstly, we discuss KPFM results from a contact surface of a copper alloy utilized in a power switch. The presence and shape of chromium grains embedded in the copper alloy are clearly visible. The contrast in the measured work function is strongly enhanced by sputtering the sample with argon ions to remove the oxide layer. Second, two different silicon carbide (SiC) devices are analysed and discussed. A calibration layer structure containing precisely defined p/n-interfaces is used to elaborate the challenges associated to KPFM and SPV measurements on semiconducting surfaces. Furthermore, a complex SiC structure of a power semiconductor device is observed by means of large area KPFM measurements. The structure is a junction barrier Schottky (JBS) rectifier-architecture [26]. We observe the termination region of the device, where highly doped p^+^-rings are embedded into a large p-type ring. These p-regions diminish the high electric field under reverse bias conditions inside the active area of the device such the the electric field gets properly terminated towards the outer rim of the device without causing unintended field peaks. Such termination regions are relatively large, therefore their detailed inspection by KPFM is only feasible due to the implemented large scan range unit.

## Experimental

The atomic force microscope (AFM) [24] developed and built in our physics department is placed in an ultra-high vacuum (UHV) system with a base pressure of *<*10^−9^ mbar. Operating the instrument under UHV condition has the advantage of a high quality factor (*Q* ≈ 30,000) due to the suppression of viscous damping and therefore increases the force sensitivity by orders of magnitude [27–28]. To analyse complex and large micro-structures a large positioning and scanning unit is necessary, under ambient conditions scan areas as large as 100 × 100 μm^2^ are available. Furthermore, the novel system provides a dedicated opto-electrical characterization using all major SPM techniques. In the following we will describe the key components of the new AFM.

### Beam deflection unit with optical excitation optics

**Figure 1:**
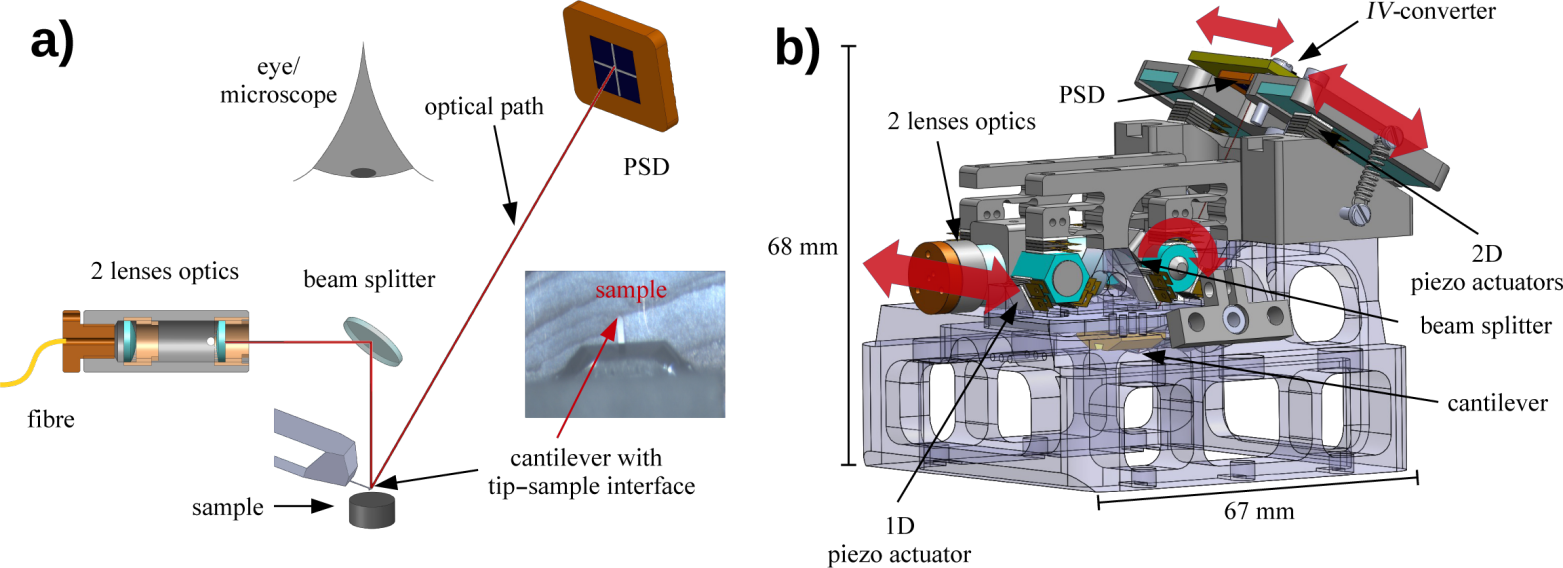
a) Schematic view of the optical path allowing good visibility from the top to the tip–sample setup. The image shows the cantilever above the sample through the beam splitter and an optical microscope. The cantilever is 225 μm long and 28 μm wide. b) Beam deflection unit with the key elements focusing optics, beam splitter and PSD with *IV*-converter. All three elements are adjustable by shear piezo actuators in order to optimally align the light beam. The directions of motion of the mobile parts are shown by the red arrows.

A fundamental design criteria in atomic force microscopy is an accurate oscillation control of the cantilever, allowing to use amplitudes in the range of 0.1–100 nm while keeping a constant tip–sample distance. Furthermore, a good visibility to the tip–sample interface is favourable in order to appropriately align the system. Beam deflection detection of cantilever oscillations is an ideal technique to fulfill these demands [29]. The light source may be placed quite far away (typically some centimetres) from the cantilever allowing for a direct optical access and the beam of light can be focused onto the free oscillating end of the cantilever. The reflected light is detected with a position sensitive diode (PSD, Hamamatsu S5980). In our case the light source is a super luminescent diode (Superlum SLD-371-HP1) with a spectral centre at 838 nm and a bandwidth of 55 nm, which is placed outside the vacuum system for reasons of thermal stability. The maximum optical power output of the diode is 7 mW. The light emitted from the diode is fed into the vacuum chamber through a single mode fibre (Fiberguide ASI4.3/125) and a custom designed optical vacuum feed-through to the focusing optics consisting of two lenses. The first lens with a focal length of 6 mm collimates the incoming light from the fibre whereas the second lens focuses the light through a beam splitter to the free end of the cantilever. The focal length is 30 mm. The optimum spot size on the cantilever free end is then the inner core diameter of the single mode fibre, in our case 4.3 μm. The optical path of this arrangement provides good visibility with an optical microscope (Olympus SZ61) from the top of the vacuum chamber through a view-port to the tip–sample interface through the beam splitter, as sketched in Figure 1a.

Figure 1b presents a computer aided design (CAD) image of the beam deflection unit. The red arrows indicate the directions of motion of the adjustable parts of the unit. To align the beam of light onto the free end of the oscillating cantilever several shear piezo actuators allow for the movement of parts within the beam deflection unit. The focusing optics is placed on two shear piezo elements in order to allow for horizontal movement and to adjust the light beam across the width of the cantilever. Additionally, the beam splitter is placed on two shear piezo elements rotating the beam splitter and therefore align the light beam along the long axis of the cantilever. The reflected light from the cantilever irradiates directly onto the PSD with an adapted current to voltage converter (*IV*-converter). The detection unit, consisting of the PSD and *IV*-converter, may be moved by three 2D shear piezo elements, adjusting the reflected light beam into the centre of the PSD. The AFM performs best when all four segments of the PSD are equally illuminated.

**Figure 2:**
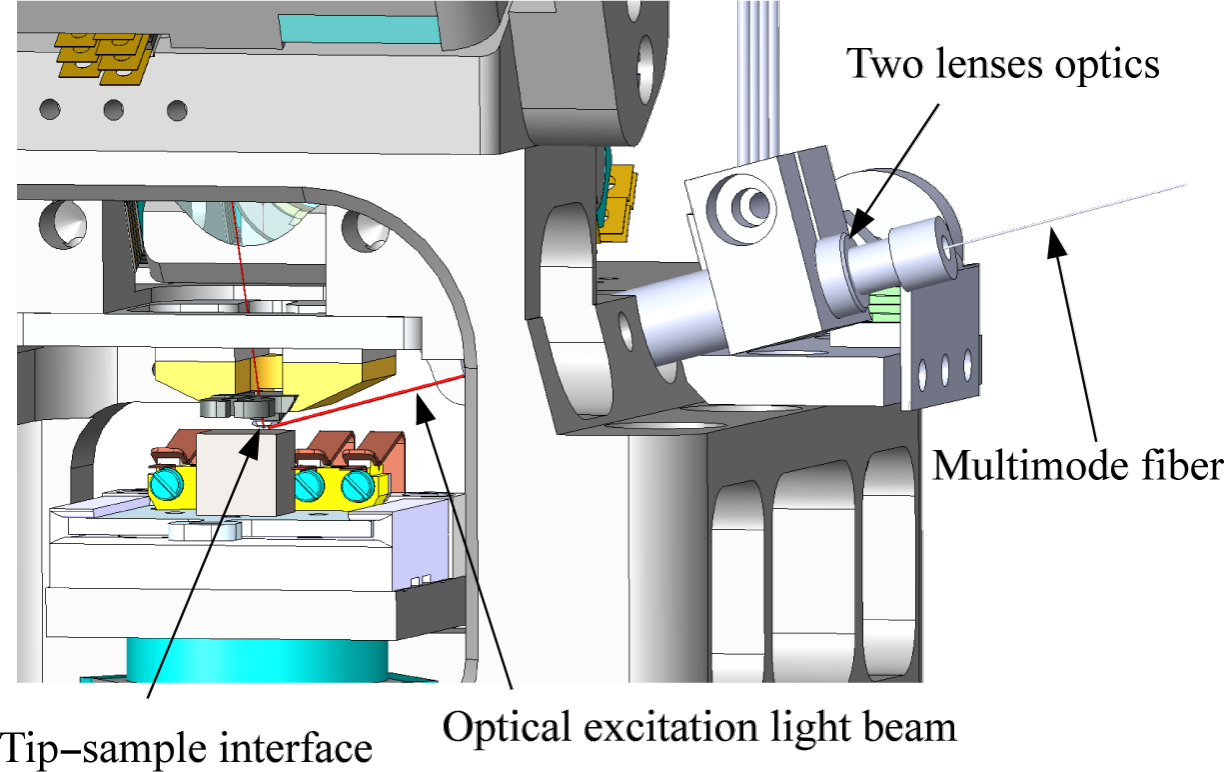
Additional two lenses optics which allows for the illumination of the tip–sample interface.

Surface photo voltage (SPV) effects enable the analysis of opto-electric sample properties and allows to minimize band bending effects at the surface of a semiconducting sample to accurately measure bulk properties by KPFM [25,30–35]. For this purpose we implemented an additional optics based again on two lenses which allows to illuminate the tip–sample interface, as shown in Figure 2. The first lens collimates the incoming light and the second focuses it onto the tip–sample interface. The optics is adjustable by piezo actuators in two directions, too. As a light source we apply an adjustable white light laser (SuperK Extreme EXW-12) with wavelengths from 450 to 2000 nm and a bandwidth ranging from 1 to 100 nm. The integrated optical power of the laser before coupling the light into the fibre of the excitation optics is about 1.2 W.

### Scan unit and coarse positioner

**Figure 3:**
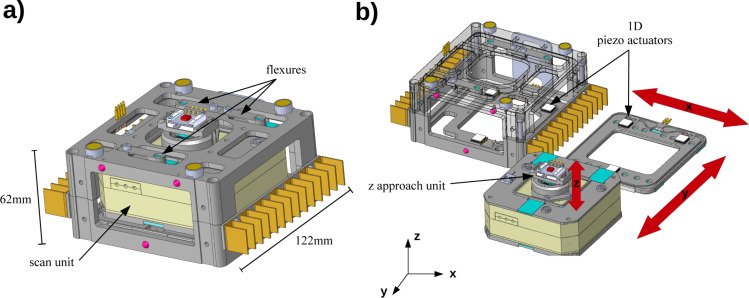
Coarse positioner and scan unit. Panel a shows the entire unit. In panel b the unit is stripped down into smaller parts. The coarse positioner consists of two stages in lateral directions, each driven by three shear piezo actuators. The stages are compressed by three flexures. The piezo step motor in the aperture of the scanner allows to approach the sample to the probe with nanoscale accuracy.

CAD images of the scan unit incorporated into the coarse positioner are presented in Figure 3. In panel a the entire unit is shown whereas in panel b the unit is stripped down into smaller parts and the stages with their directions of motion indicated by the red arrows are visible. The desired scan range of 100 μm in lateral (*x* and *y*) directions and 25 μm in vertical (*z*) direction is realized by a commercial closed-loop scanner (nPoint, NPXY100Z25A). The spatial resolution of the scanner is in the sub-nanometre regime for both lateral and vertical directions according to the specifications provided by the manufacturer. Atomic resolution in vertical direction is demonstrated later in our article (see Figure 6). The scan unit is placed on two custom designed piezoelectric stages, which allows for a travel range of 12 mm in lateral directions. A stage consists of three 1D shear piezo actuators and two sapphire sliders. Three flexures compress the two stages together. The scanner has an aperture of 38 mm in which an additional piezo actuator is located with a travel range of 20 mm in vertical direction, used for coarsely approaching the sample to the cantilever tip. All piezo elements are driven with a custom designed controller generating saw tooth voltages with amplitudes ranging from 0 to 400 V and frequencies up to 1 kHz.

### Damping system

**Figure 4:**
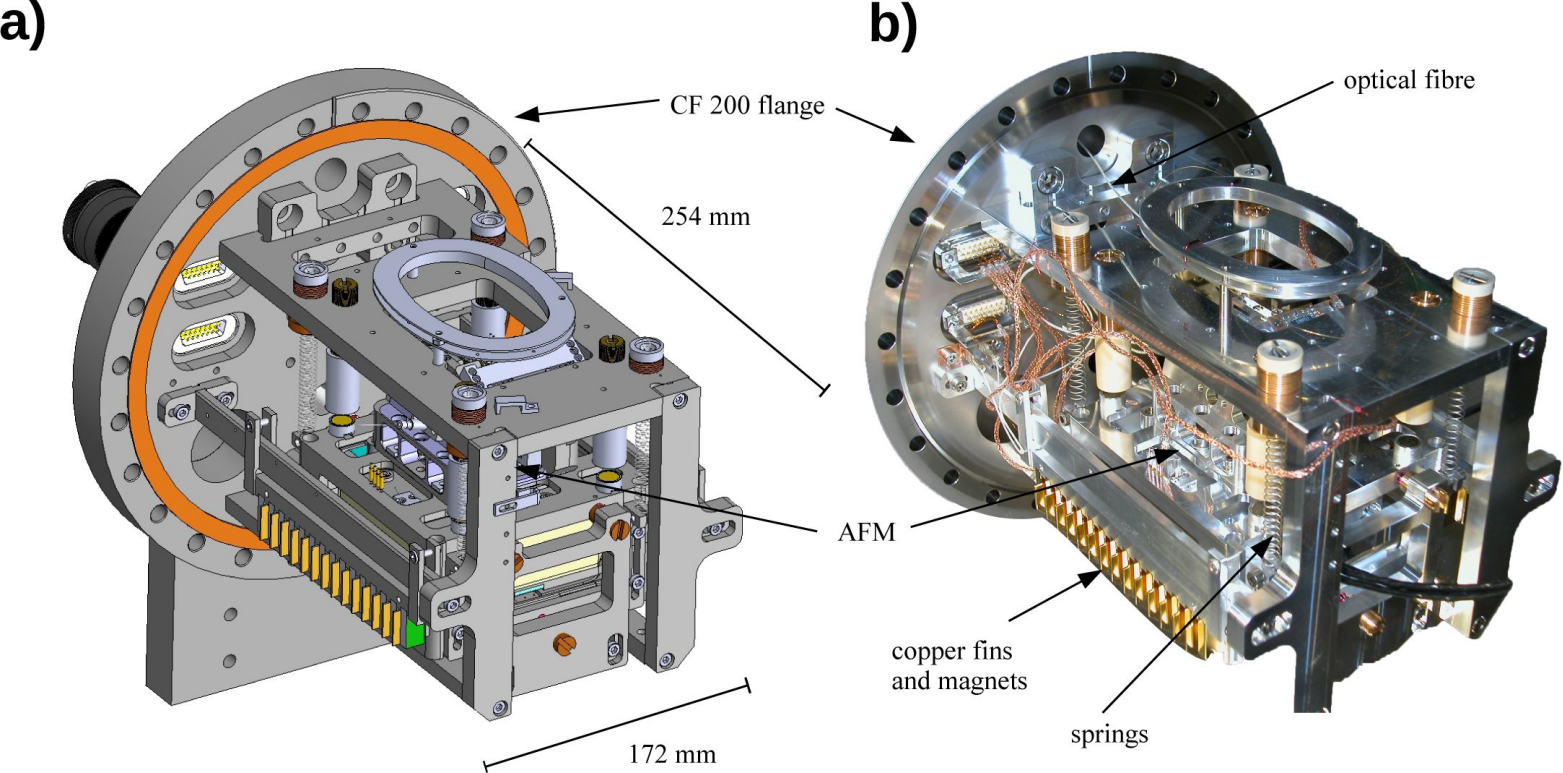
The atomic force microscope is assembled on a CF200 flange with four tension springs. Copper fins and magnets serve as an eddy current damping system to suppress vertical vibrations. In panel a, a CAD image is illustrated, whereas panel b shows a photography of the microscope.

Figure 4 shows the CAD (panel a) and the photographic (panel b) image of the complete AFM system. The beam deflection and scanner units are mounted together on top of the coarse positioner and represent the heart of the microscope. The system is attached to a CF200 flange and is suspended on four tension springs. Strong magnets (NdFeB magnets) with a horizontal field of about 0.5 T and copper fins serve as an eddy current damper for vertical vibrations. At each corner of the instrument magnets are placed, which can be adjusted in height. The magnets are oriented such that they attract and therefore reduce the weight force of the instrument, allowing for the utilization of softer springs and hence reduction of the resonance frequency of the damping system.

### Electronics and SPM software

The current signals from the PSD are converted into voltage signals by a custom designed *IV*-converter with a bandwidth of 3 MHz, which is located on the rear side of the PSD in UHV. The signals from the converter pass through an electrical vacuum feedthrough to a custom designed electronics that computes the sum and differences of the four diode segment signals and amplifies them. All cables are carefully shielded by a copper mesh to avoid capacitive cross talk between the individual PSD signals, the piezo and electrical excitation, and the scanner signal lines. Several separated electrical UHV feedthroughs are implemented for the sample bias voltage, the piezo excitation, the PSD signals, the scanner, and the coarse motion, respectively.

To control the instrument, a commercially available electronic equipment is used (SPECS, Nanonis). For bimodal measurement techniques, such as KPFM and SCFM, two independent phase lock loop (PLL, Nanonis OC4) circuits are necessary. The SPM software consists of several modules allowing to control the PLLs, the beam deflection alignment, the Kelvin controller, the coarse positioner, and the scan unit. The closed loop scanning system includes a control unit with PID-controllers for each axis. The scan area is controlled with analog signals from the SPM electronics.

## Results and Discussion

To distinguish between different materials in metallic or differently doped regions in semiconductors several scanning probe microscopy methods are implemented in our novel microscope. KPFM measures the difference of the contact potential difference (CPD) between the tip and the sample by applying a dc voltage *V*_CPD_ to nullify the electrostatic force acting between them [9,36]. A very sensitive way to measure, separate, and compensate the electrostatic forces is the so-called amplitude modulated KPFM (AM-KPFM) which uses the second eigenmode of the cantilever [17,37]. By applying an ac voltage *V*_ac_ to the tip–sample system exactly at the second eigenmode, the cantilever starts oscillating at this frequency while the amplitude depends linearly on the dc potential drop (*V*_dc_ − *V*_CPD_) between tip and sample. By then applying a dc compensation voltage *V*_dc_ the amplitude can be minimized and the contact potential difference *V*_CPD_ can be determined. While this method works fine for metallic surfaces special care has to be taken on semiconducting or insulating surfaces. The main challenge arises from the fact that the tip–sample capacitance is no longer independent of the applied voltage such that higher harmonic contributions between the individual PSD signals, influence the measurements [22]. Furthermore, band-bending effects due to surface defects and the applied ac voltage may change the measured *V*_CPD_ [38–39]. Hence KPFM is an ideal experimental technique to visualize electronic properties of all kind of surfaces, however, with the aforementioned straight forward interpretation of the results for metallic materials with different work functions as for example in an alloy.

### KPFM of a Cu/Cr alloy

**Figure 5:**
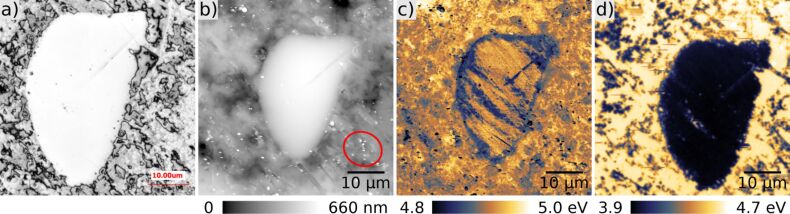
A chromium grain embedded in a polycrystalline copper alloy. a) Measured with a confocal laser microscope. b) Topography and c) KPFM image of the grain before sputtering. d) KPFM image after two sputter cycles for 10 min each with Ar^+^-ions and a voltage of 1 kV. Small tip changes, as highlighted by the red circle in panel b, are not influencing the work function measurement sustainably. The work function difference between copper and chromium measures ΔΦ = 720 meV. Scan size: 50 × 50 μm^2^.

Figure 5 presents a copper alloy with incorporated micrometre-sized chromium grains used in high voltage power switches. Each switching process results in a melting of the contact surface and after several hundred events in a degradation of the device properties. Therefore, the chemical and structural properties of these surfaces are of major interest. Since the melting zone is typically macroscopically sized a feature of interest, here a chromium grain, can be localized by a confocal laser microscope (Keyence VK-X100K/X200K) in air (Figure 5a). After transferring the sample into the UHV system, the same grain is approached with the coarse positioner and KPFM experiments are performed on the grain and its surroundings. The large arithmetic average roughness of *R*_a_ = 168 nm over a scan range of 50 × 50 μm^2^ results in a time of 6 h to acquire these images in a reasonable resolution and stability. For that reason very stable conditions, e.g., in temperature, are required. For these measurements a n^+^-doped silicon cantilever with a PtIr-coated tip (Nanosensors, PPP-EFM) was used. The cantilever has frequencies of *f*_1st_ = 71 kHz for the first and *f*_2nd_ = 447 kHz for the second eigenmode. In the topography (Figure 5b) as well as in the *V*_CPD_ (Figure 5c) images the chromium grain is clearly observable. The grain seems to be covered by a residual layer partly smearing out the CPD contrast. The PtIr-coated tip is most probably contaminated by a metal oxide cluster (CuO or CrO) due to slight tip–sample contacts before the measurements, such that the work function is around Φ_tip_ = 5 eV [40]. Also in Figure 5b small tip changes are visible as stripes (some of them indicated by the red circle), however, the work function measurement is not influenced sustainably. After sputtering the surface with Ar^+^-ions twice for 10 min with a voltage of 1 kV, the contamination layer is removed and the contrast in the CPD reflects the unperturbed work function values as presented in Figure 5d. The difference in the CPD between copper and chromium is about 0.7 V, while the work function of the chromium grain is reduced to approximately Φ_Cr_ = 3.9 eV and the one for the polycrystalline copper to Φ_Cu_ = 4.6 eV. Both values are in excellent agreement with other experimental values [40]. The new large-area AFM allows not only to image the structural modifications of surfaces but also to acquire quantitative electronic information of the specimen with nanometre-scale resolution.

### SiC calibration structure

However, many samples of interest are semiconductor surfaces involving various doping concentrations and even cross-sections of interfaces [41–42]. Such measurements are influenced by surface band-bending effects induced by either intrinsic surface defects, adsorbates, interface states and last but not least by the doping concentration. Since KPFM is a non destructive, surface sensitive technique, e.g., compared to SSRM, information on bulk properties have to be extracted from the surface sensitive information. Several approaches have been applied in recent years for this purpose, e.g., avoiding surface defects by special preparation techniques, depositing additional known termination layers or using additional measurement techniques to separate bulk from surface information as the aforementioned SPV measurements. Recently, the presented SPM system was applied to the analysis of complex SiC structures [43].

To understand the contrast mechanism in KPFM, measurements on a SiC calibration sample have been performed to elucidate the major requirements for getting qualitative and quantitative results. Generally, high p^+^-doped regions have Fermi-levels *E*_F_ approaching the upper edge of the valence band *E*_V_ and hence have a higher work function Φ than lower doped p-areas, where the Fermi level is below the centre *E*_i_ of the valence *E*_V_ and conduction band *E*_C_. Anyhow n-doped areas have anyway a lower work function than p-doped areas. However, cross-section measurements have already shown, that direct measurements of the Fermi-level position on SiC samples is strongly affected by surface preparation and material properties [44].

**Figure 6:**
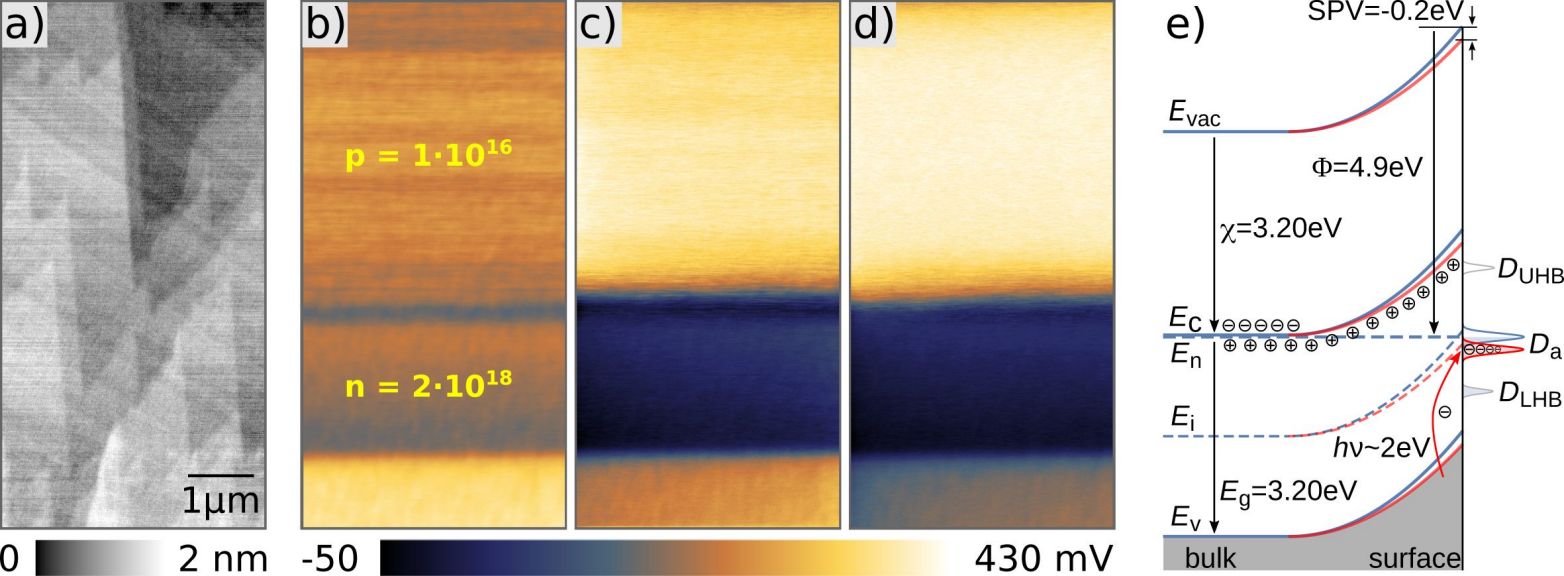
a) Topography, b) dark KPFM and c) 30% laser-power illuminated (470–480 nm and a maximum power of 50 mW) KPFM images of a SiC p/n-junction. The scan size is 4 × 8 μm^2^. d) 100% laser- power illuminated KPFM image. e) shows a schematic band diagram of the surface band bending as well as the characteristic energies for the n-doped SiC sample including known defect states in dark (blue) and under illumination (red). Under illumination electrons are excited to the acceptor-type defects *D*_a_ and the band-bending is reduced, details are explained in the text.

Figure 6 shows a KPFM measurement of a SiC calibration sample consisting of a 2 μm thick nitrogen-doped n-type (*N*_N_ = 2 × 10^18^ cm^−3^) followed by a 4 μm thick aluminium doped p-type (*N*_Al_ = 1 × 10^16^ cm^−3^) epitaxially grown SiC layer stack on top of a highly doped n-type SiC substrate. The cross section of the sample was cleaved right before introducing it into vacuum and the topography (Figure 6a) shows steps running across the differently doped areas. The steps have atomic character, as the difference in height between adjacent steps is 0.2–0.4 nm emphasizing the performance of the developed AFM. The arithmetic average roughness *R*_a_ has a value of 0.45 nm. However, traces of the differently doped layers are not directly visible. The simultaneously recorded CPD image is shown in Figure 6b. In these experiments the bias voltage was applied to the sample while the tip was grounded and the used ac voltage in KPFM was 500 mV tuned to the second eigenmode of the cantilever. The data are measured with a n^+^-doped silicon cantilever coated with platinum/iridium (Nanosensors, PPP-NCLPt) with eigenfrequencies of 150 kHz for the first and 950 kHz for the second eigenmode. The contrast of the *V*_CPD_ shows weakly the expected three interfaces but with a much smaller potential difference as one could expect. The variation between the p-type area and the n-type area is expected to be close to the electrical bandgap of SiC which is in the range of *E*_g,SiC_ = 3.25 eV. Or, more accurately, it should correspond to the built-in potential *V*_b_ which one could ideally expect across the interface. The theoretical *V*_b_ calculates as [45]:

[1]
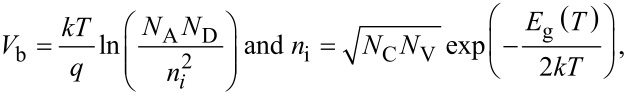


where *k* is the Boltzmann constant, *T* the temperature, *q* the elementary charge, and *N*_A_ and *N*_D_ the doping concentrations for the p- and n-type materials, respectively. The intrinsic charge carrier concentration can be calculated to be *n*_i_ = 9.65 × 10^−9^ cm^−3^ by using the temperature-corrected values (*T* = 300 K) for the band gap *E*_g_ = *E*_g,0_ − 6.5 × 10^−4^·*T*^2^/(*T* + 1300) = 3.25 eV and the effective density of states in the conduction (*N*_C_ = 3.25 × 10^15^·*T*^3/2^ = 1.69 × 10^19^ cm^−3^) and valence band (*N*_V_ = 4.8 × 10^15^·*T*^3/2^ = 2.49 × 10^19^ cm^−3^) [46]. This leads to *V*_b_ = 3.0 eV, which is a reasonable value taking into account the band gap of 4H-SiC. With a theoretically determined electron affinity of χ = 3.1–3.2 eV [47–48] one gets work function values for the p- and n-type areas of Φ_p_ = 6.2 eV and Φ_n_ = 3.2 eV, respectively. Assuming a work function of Φ_tip_ = 4.28 eV for the PtIr-coated tip [49], results in work function values of the SiC cross-section of Φ_SiC_ = 4.5–4.7 eV indicating a Fermi-level pinning at the surface at an energy around mid band gap (*E*_i_ = 4.6 eV).

For SiC it was already observed before, that the measured work function seems to be largely independent of the doping concentration indicating a well defined Fermi-level pinning at approx. 4.6 eV due to intrinsic surface-state bands [50]. Furthermore, different surface orientations show variations of the work function of 250 mV and a large statistical variation of the measured values due to different surface preparation techniques is frequently reported [51]. However, a very nice overview on electronic properties of SiC surfaces and interfaces is given by T. Seyller [52]. The electronic structure of SiC surfaces suffers from a strong electron correlation induced by a Mott–Hubbard metal–insulator transition [53] due to a half-filled and hence metallic band arising from dangling bonds. More refined studies employed a 2D Hubbard model indicating that the energy levels of the SiC surface consist of a filled band and an empty band, separated by a Hubbard gap of 1.6 eV. A pinning of the Fermi level was also observed by STM studies differing only by about 200 mV between p- and n-type doped SiC [54]. SiC was found to be in the transition between strong and no Fermi-level pinning which could also be tuned by passivation of the surface states with, e.g., hydrogen [55]. Furthermore, a large density of electrically active defects just below the conduction band of the polytype 4H-SiC has been reported to appear at interfaces and maybe also affecting the electronic structure at surfaces [56]. Especially carbon clusters are responsible for donor states in the lower part of the bandgap as well as a continuum of donor- and acceptor-type states in the central part of the band gap.

**Figure 7:**
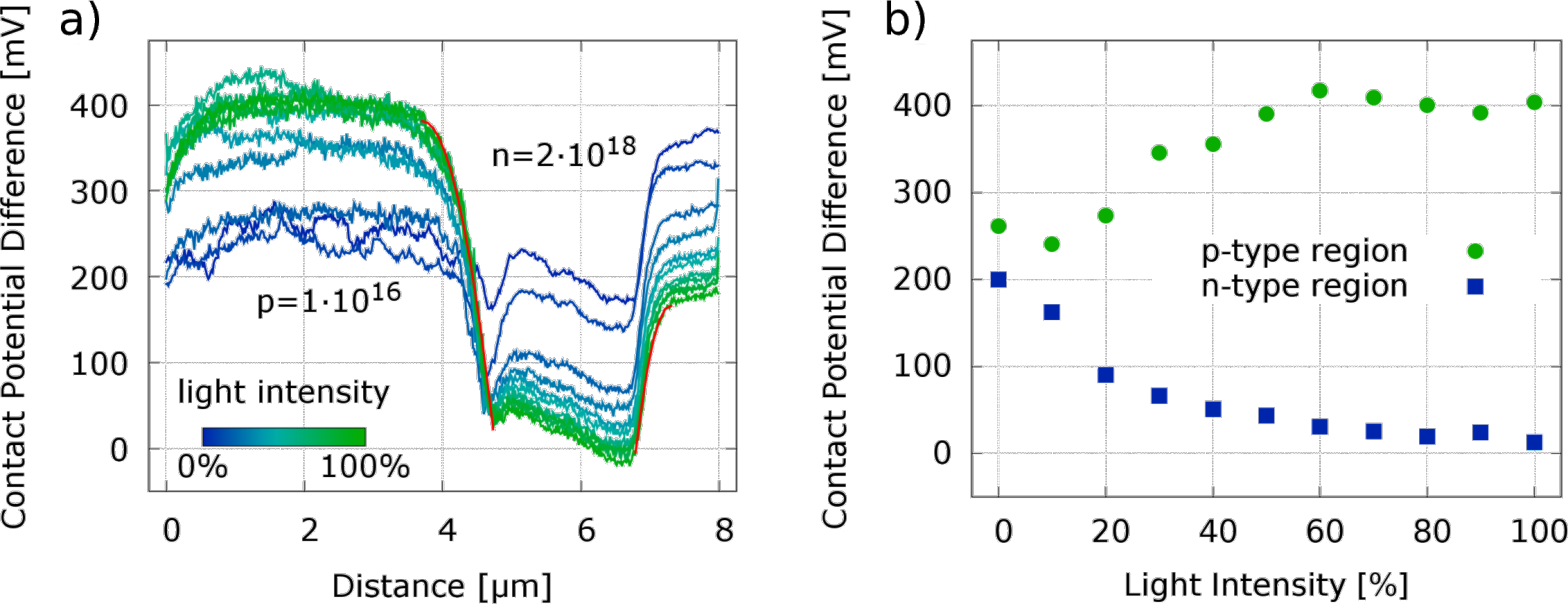
Panel a shows sections across the SiC p/n-junction (Figure 6) extracted from images taken at various light intensities. The data were averaged over five lines taken along the middle of the scan area. The averaged CPD values in the p- and n-type areas approach constant values under illumination as shown in panel b. In red are shown fits of the SCR regions of both junctions from the 100% illuminated sample calculated through Equation 2.

Thus, the measurements presented here fit perfectly in this picture of a strongly defect and adsorbate-influenced Fermi-level pinning of the SiC surface. A well-known technique to address and quantify the influence of surface defects in semiconductors are surface photo voltage (SPV) measurements. Charge carriers are excited by an incident photon flux and the generated electron–hole pairs are reducing the surface band bending depending on the illumination intensity and energy even until flat-band conditions. To demonstrate the impact of SPV, we illuminated the SiC p/n-junction with laser light of 470–480 nm and a power of maximum 50 mW. An increase of the CPD contrast is clearly observable by comparing in Figure 6 the KPFM measurements without (panel b) and with (panels c and d) laser illumination with 30% and 100% intensity, respectively. However, the achieved maximum difference between the n- and p-type areas of 430 mV is still far away from the expected built-in voltage. In Figure 6e a schematic band diagram shows the influence of the surface band bending as well as the characteristic energies for the n-doped SiC sample. The surface defect states are placed at energies as determined by T. Seyller [52]. *D*_UHB_ and *D*_LHB_ are the upper and lower Mott–Hubbard bands, respectively, located at fixed energies with respect to the Fermi level. In an n-type semiconductor only the acceptor type defects *D*_a_ located at the centre of the band gap are responsible for the observed surface band bending. A similar scheme with a downward band bending and donor-type defects is valid for the p-type case. Under illumination electrons are excited from the valence band to the acceptor-type defects at mid bandgap. Consequently, the charge carrier density at the surface is changed and the band bending is reduced, however, only until all acceptor type defect states are filled up. The same process holds true for the p-type SiC where a reduction of the band bending until all donor-type defect states are filled up by holes is expected. Therefore, the measured change of *V*_CPD_ under illumination corresponds to the width of the defect distribution in the centre of the band gap. To fully diminish the surface band bending in SPV measurements higher light energies overcoming the band gap of SiC are necessary. Figure 7a shows line section data extracted across the SiC p/n-junctions as presented in Figure 6 for various light intensities ranging from 0 to 100%. The evolution of the average CPD of the p- and n-type area with the light intensity is plotted next to it (Figure 7b) and shows an increase of the CPD by 200 mV for the p-type area and a decrease by the same amount for the n-type area. Both values are saturating with increasing light intensity.

Some more detailed features can be observed in the line sections shown in Figure 7a. At the p/n-junction a dip in the CPD can be observed which vanishes under illumination. This might indicated that the interface states are already fully charged before illumination inducing a dip in the surface potential. Furthermore, the linear decrease of the CPD within the n-type layer that is unaffected by the illumination might be related to a constant electric field between the p-type layer and the SiC substrate. More quantitative information can be extracted from the transition of the CPD into the p-type area, which can directly be related to the space charge region (SCR) that develops due to the interdiffusion of oppositely charged carriers at the interface. Following the arguments in chapter 2.2.1 of [45] for a one-sided abrupt junction (p^+^/n or p/n^+^) the potential distribution across the junction can directly be related to the build in potential (*V*_b_) and the width of the SCR (*W*):

[2]
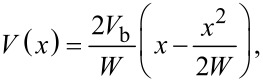


where *x* corresponds to the distance from the p/n-junction. The total width of such an abrupt SCR is given by:

[3]
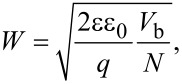


where *N* is *N*_D_ or *N*_A_ depending on whether *N*_A_ *>> N*_D_ or vice versa and ε = 9.66 is the relative permittivity of SiC. For the case discussed here most of the depleted SCR will be located in the lowly doped p-type area and a least-square fit of the data by Equation 2 at maximum illumination results in a SCR width of *W* = 880 nm and a built-in voltage of *V*_b_ = 270 mV as shown by the left red curve in Figure 7a. The second fit from the transition of the n-type area to the substrate results in a width of *W* = 500 nm and a built-in voltage of *V*_b_ = 170 mV. As expected the built-in voltage is much smaller than the theoretically expected value calculated by Equation 1, but the SCR width is at least in the same order of magnitude as the value *W*_theo_ = 570 nm that is analytically calculated through Equation 1 and Equation 3 [57]. A much longer decay of the surface potential was also observed by M. Gao et al. in locally resolved secondary electron emission measurements across a SiC p*/*n-junction [44]. They attributed the increase of the SCR to near-surface dopant reduction induced during sample surface preparation which in our case would result in a effective doping concentration at the surface of the cross section of *N*_Al,eff_ = 4.1 × 10^15^ cm^−3^ utilizing Equation 3. In the case of the n-type substrate we get an effective doping concentration of *N*_N,eff_ = 1.3 × 10^16^ cm^−3^.

### SiC JBS device structure

Finally, we applied the technique to analyse the electronic structure of a complex SiC power semiconductor device. SiC material properties enable devices compatible with higher voltages and operating temperatures compared to traditional Si-based architectures for power electronic switches and rectifiers [58]. However, the reduction of the Schottky barrier height as well as tunneling processes are still limiting the voltage and efficiency of SiC Schottky barrier diodes. An alternative approach for such devices is the so-called junction barrier Schottky (JBS) rectifier-architecture, where highly doped p^+^-regions are embedded into the active device area to shield the Schottky contact from high electric fields and to handle surge-current events at the same time [59–60]. The implantation of dopants as well as the electronic properties of these embedded shields is a key property and needs sophisticated characterization [57].

**Figure 8:**
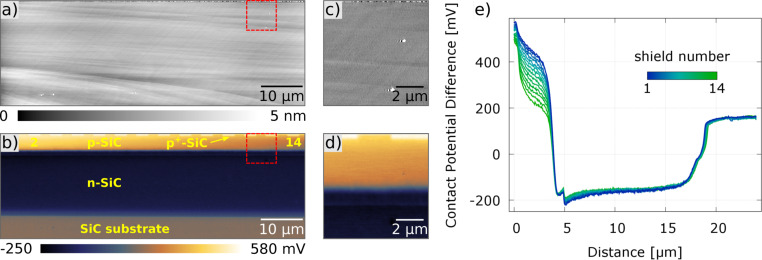
Simultaneously acquired topography (a) and CPD (b) images of a silicon carbide JBS structure. The scan range is 70 × 24 μm^2^ with an arithmetic average roughness of *R*_A_ = 5 nm. Differently doped regions of the SiC sample are clearly identified in the KPFM-signal. A zoom of the topography and the CPD data marked by the dashed square is shown in c) and d), respectively. Line sections across the different interfaces through each center of a p^+^-doped area are shown in graph e). The shields (width 2.8 μm) are numbered from left to right and have a periodicity of 4.9 μm.

The KPFM experiment presented in Figure 8a and Figure 8b is an example of a large area 70 × 24 μm^2^ cross section from such a SiC power device determined also by the aforementioned AM-KPFM technique and a PtIr-coated Si cantilever in dark. The device was cleaved in air and then transferred into the UHV system, such that a homogeneous distribution of surface defects was expected. The topography (Figure 8a) shows no major contrast, whereas the simultaneously acquired CPD (Figure 8b) clearly distinguishes between the n-, p- and p^+^-doped regions even without illumination. The arithmetic average roughness of *R*_A_ = 5 nm over the entire scan range is astonishing. The measuring time for this image was 2 h and 45 min and the used ac excitation voltage was only 100 mV to avoid any tip-induced band bending effects. In the light of the discussion before the detectable CPD contrast indicates a weaker Fermi-level pinning at the centre of the band gap. However, still the overall contrast of roughly 700 mV does not represent the actual built-in voltage of the interface. The marked areas have been enlarged in Figure 8c and Figure 8d to visualize the JBS-structure of the device. The p^+^-doped shields within the p-type layer are clearly visible as bright areas on top of the CPD image and the n-type areas at the bottom. Taking again a line-section across the interfaces allows to extract more details. Figure 8e shows several vertical line sections across the complete sample always located at a centre of a p^+^-doped shield. In total 14 curves starting from the left to the right are presented. Beside the variation between the differently doped areas the most eye-catching feature is the pronounced change of the potential in the p-type SiC layer, while at the same time neither the potential in the substrate nor in the n-SiC is changing noticeably. Also an edge effect on the KPFM signal can be excluded since that should be affecting all other layers, too. Since the sample was contacted homogeneously at all sides also the influence of an external inhomogeneous bias distribution can be neglected, as well as an inhomogeneous surface defect distribution, which should also be apparent at the other layers. Assuming the SiC substrate has the literature work function of Φ_SiC_ = 4.6 eV, the n-type region has a work function of Φ_n−SiC_ = 4.2 eV and the p^+^-type region of 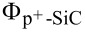 = 5.0 eV, which are in reasonable agreement to published values [61].

**Figure 9:**
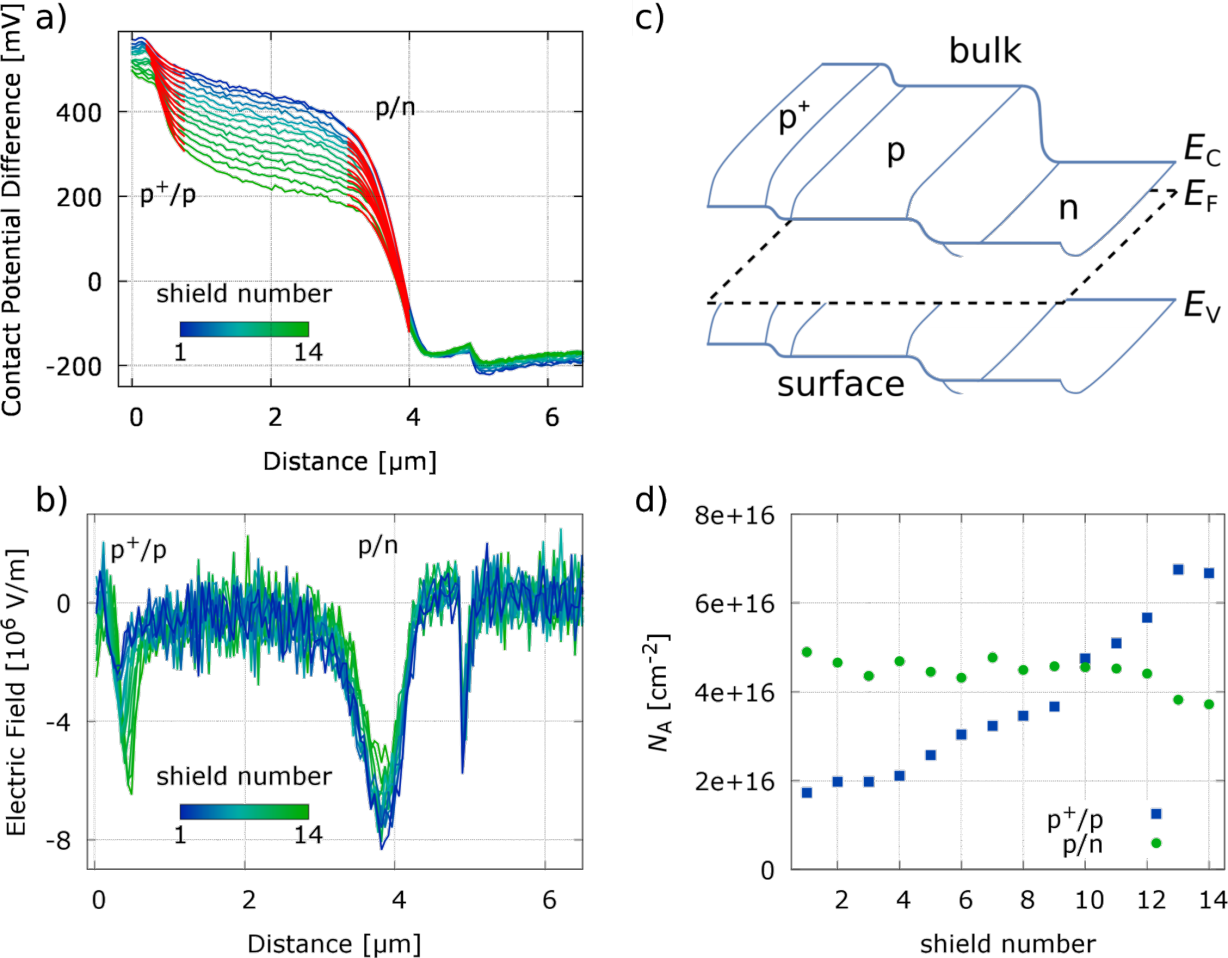
a) Close view of the line sections from Figure 8e at the top layer of the structure, together with least-square fits through Equation 2 of the p^+^/p- and p/n-interface in red. b) Calculated electric field from panel a again from shield 1 to 14. The determined built-in voltage *V*_b_ and SCR width *W* from the fits were used to calculate the acceptor concentration *N*_A_, as presented in panel d, of the p-type layer at the 14 different positions for both the p^+^/p- and the p/n-interface. c) shows a schematic view of the complex band bending features expected at the surface of the SiC cross section.

Figure 9 shows the results of a detailed analysis of the p^+^/p- and the p/n-interfaces again dependent on the position along the sample. The length of the line sections corresponds to the scan area presented in Figure 8c and Figure 8d. In Figure 9a and Figure 9b the CPD as well as least-square fits by Equation 2 of the p^+^/p and the p/n-interfaces and the corresponding electric field *E* = d*V*_CPD_/d*z* is shown. This field corresponds to the lateral effective field across the respective junction and should not be confused with the perpendicular field between tip and sample, which is minimized by using KPFM. Starting at around 4.5 μm the n-type area is located showing a zero electric field. The visible kink in the CPD and corresponding spike in *E* in this area is not changing with position and seems to be related to a defect layer introduced during the growth process of the sample, however, details are unknown. The p/n-junction at around 4 μm exhibits a maximum electric field of *E*_p/n_ = −8 × 10^6^ V/cm, which is slightly decreasing with the position (shield number). However, the curvature neither on the n- nor on the p-side seems to be strongly influenced by the position so that also the least-square fit, as shown in Figure 9a, results in a constant SCR width of *W*_p/n_ = 840 nm and a slightly decreasing built-in voltage of *V*_b,p/n_ = 340–240 mV. Within the p-layer a linear drop of the CPD from the p^+^ to the n-layer is observed which is constant with an averaged electric field strength of *E*_p_ = −0.5 × 10^6^ V/cm at all 14 positions. The p^+^/p-interface shows, however, two very distinct effects, the electric field drop across the junction increases with increasing shield number while also the width of the SCR is changing as can be seen by the width of the potential drop (Figure 9b). The results from the least-square fit by Equation 2 show an increase of the built-in potential 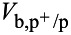 from 400 to 500 mV as well as a clear decrease of the SCR width 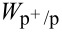 from 1600 to 900 nm. As discussed before KPFM measurements can only be used under distinct surface conditions to predict bulk information from measured surface properties. Here we have a complex SiC structure influenced by some surface defects and states within the band gap, which induce a laterally homogeneous downward band bending for p-type and opposite for n-type material. Therefore, Equation 2 can be used to evaluate measured surface potentials to get at least surface relevant information. Figure 9c shows a sketch describing the used band structure and the surface effect. However, such effects are not only limited to surfaces but may also occur at interfaces impacting device properties. Assuming that the p^+^ and the n-type layers have a higher acceptor and donator concentration than the p-type layer the acceptor concentration *N*_A_ of the p-type layer can be calculated via transforming Equation 3 to:

[4]
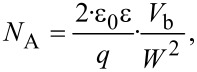


resulting in an effective surface-doping concentration for each position and interface as presented in Figure 9d. The value of *N*_A_ = 4 × 10^16^ cm^−2^ is reasonable and under the assumption that only the built-in voltage changes from the surface towards the bulk one calculates a bulk concentration of *N*_A_ = 4 × 10^17^ cm^−2^ for an estimated built-in voltage of 3 V. Therefore, the change of the surface potential can directly be associated with a change in the doping concentration of the p-SiC layer.

## Conclusion

A novel atomic force microscope with a large scan area is operated under UHV conditions at room temperature. The instrument is ideal to analyse devices, either conducting or semiconducting, which are the major building blocks of power devices. On the conducting sample we perform KPFM measurements, showing different components in a copper alloy used as contact in power switches. On a SiC calibration structure the differently doped areas were clearly distinguished in the KPFM-signal, whereas the topography did not reflect the different areas, as expected. Surface photo voltage induced reduction of the band bending at the surface is demonstrated on these SiC p/n-junctions illuminated with different laser power levels. The gained knowledge is applied to the analysis of a complex SiC JBS cross section and limitations and challenges of the KPFM technique have been discussed.
